# Upper Extremity Axillary Loop Grafts: An Opportunity in Hemodialysis Access

**DOI:** 10.5812/numonthly.5056

**Published:** 2012-12-15

**Authors:** Jalaladin Khoshnevis, Mohammad Reza Sobhiyeh, Saran Lotfolahzadah, Fatemah Hoseinzadegan Shirazi, Amir Hosein Jalali

**Affiliations:** 1 Department of Vascular Surgery, Faculty of Medicine, Shahid Beheshti University of Medical Sciences, Tehran, IR Iran; 2 Department of Surgery, Faculty of Medicine, Shahid Beheshti University of Medical Sciences, Tehran, IR Iran

**Keywords:** Renal Dialysis, Transplants, Polytetrafluoroethylene

## Abstract

**Background:**

Dialysis vascular access complications are considered as significant causes of morbidity in chronic hemodialysis patients.

**Objectives:**

The aim of the present study was a comparison of axillary loop and straight grafts patency and its complications in hemodialysis access.

**Patients and Methods:**

In this cohort study conducted at Shahid Beheshti Medical University, 77 patients who underwent placement of loop or straight access grafts were included. Demographics, primary and secondary patency rates and complications like thrombosis, infection, bleeding, steal syndrome and other complications were compared in these two groups. The collected data was analyzed by chi-square test, t-test, and logistic regression.

**Results:**

Primary patency rate in straight and loop groups after 1 month were 88.9% and 92.3% respectively (*P* = 0.721), and after 24 months were 31% and 55.5% respectively (*P* = 0.058). Secondary patency rate in straight and loop groups after 3 months were 75.6% and 92.3% respectively (*P* = 0.189), and after 24 months were 37.9% and 66.7% respectively (*P* = 0.044). The frequency of complications were the same among two methods of graft replacement and mal incidence of thrombosis, infection, delayed infection, pseudoaneurysm formation and steal syndrome occurrence ultimate graft failure and venous hypertension were not significantly different (*P* > 0.05).

**Conclusions:**

Polytetrafluorethylene (PTFE) vascular graft seems to be an appropriate vascular access and is a promising alternative when upper extremity arteriovenous fistulas cannot be constructed. Additionally, there was no significant difference between the two groups for complications and early patency, but late patency was improved in loop group. More study is necessary for a conclusive assessment.

## 1. Background

Renal failure and its complications are among major dilemmas in health care and treatment worldwide, resulting in one of the crucial causes of morbidity and mortality (1). Recently the prevalence of end stage renal disease has significantly increased and has reached 49.9 in 100/000 person during last decade which is explained by a raised life expectancy and the increased prevalence of several underlying diseases such as diabetes mellitus (2). Placement of a suitable vascular access is very important (3).

Hemodialysis efficacy depends on vascular access function which is divided in two subgroups; permanent access by arteriovenous fistula or arteriovenous graft as well as temporary access by cuffed catheter (3). Despite the definite advantages of areteriovenous fistula, due to lack of suitable veins, the fistula becomes of use immediately after its placement or a short while after (4).

In 1976, polytetrafluorethylene (PTFE) prosthetic grafts were innovated (3). PTFE grafts have notable advantages, including lower thrombogenicity, higher resistance to repeated punctures, availability of different sizes, and yields favorable arteriovenous access (5, 6). But given the problem of the cost of vascular surgery and vascular grafts, maintaining graft patency is crucial, to the extent that it prevents recurrent surgery for obtaining new vascular access (7-9). Nowadays, several methods have been introduced that facilitate axillary loops and straight grafts in the upper arm (10).

## 2. Objectives

There is no comparison study that denotes patency rate in the abovementioned two methods. This study plans to assess the efficacy and complications of upper arm curved grafts versus the straight ones.

## 3. Patients and Methods

In this historical cohort study, conducted at Shohadaie-Tajrish Hospital (Shahid Beheshti Medical University, Tehran, Iran), 77 patients who underwent placement of loop or straight vascular access grafts were included. They were all above 18 years-old, affected by chronic renal failure, and referred to the Shohadaie-Tajrish Vascular Clinic during 2004-2010. Placements of other vascular access such as arteriovenous fistula were impossible. Those with incomplete clinical documents were excluded from the study.

From a total of 77cases, 56 patients received straight grafts, while 21 patients received loop grafts, and all grafts were placed in the upper arm. All surgeries were carried out by a single vascular surgeon based on a standard technique with a polytetrafluorethylene graft, commercially named Gore-tex. The length of the graft was 20 centimeters for the straight type versus 40 cm for the curved one. All the patients were started on hemodialysis via the new graft at least 2 weeks after the procedure.

Age, sex, infection rate, bleeding rate, false aneurysm formation rate, steal syndrome rate, venous hypertension rate, hypertension, diabetes mellitus, aspirin consumption, duration of graft placement to first thrombosis and thrombosis formation rate in the graft were all recorded. Primary patency was defined as the time interval of graft patency among the arterial and venous system from placement time until the first experience of the graft being out of use without any intervention regardless of its causality, whereas secondary patency was described as the period of time from graft implantation to its closure despite all efforts.

The patients were followed up in a duration of 1, 3, 6,10,18,24 months and the assessment of graft's patency was carried out at the same time. Physical examination and presence of thrill palpation or bruit auscultation in the site graft placement were considered as an assessment of graft patency and complications. Follow-up visits were discontinued when the patients passed away, the follow-up time was completed or if the grafts were out of order. The collected data was analyzed by the SPSS software using the chi - square test, t - test. To eliminate possible confounding factors and their effects, Logistic Regression was used.

## 4. Results

There were 17 males (30.35%) and 39 (69.65%) females (mean age 56.3 ± 13.3 years) in the straight graft group versus 7 males (33.33%) and 14 females (66.66%) (mean age 53.1 ± 11.1) in the curved graft group.

In the straight graft group, 18 patients (32.14%) received grafts with 8 millimeters diameter while 38 patients (67.85%) received 6 millimeter ones. Nine patients (42.85%) in the curved graft group received 8 mm grafts versus 12 patients (57.14%) who received 6 mm ones. The proportion and frequency of diabetes mellitus and hypertension is elucidated in Table 1 which didn’t show a significant difference among the two groups.

**Table 1:** Proportion and Frequency of Diabetes Mellitus and Hypertension Between Straight and Loop Group’s

	Diabetic No (%)	Without-Diabetic No (%)	Hypertension No (%)	Without-hypertension No (%)
**Straight**	20 (32.71)	36 (64.28)	32 (57.14)	24 (42.85)
**Loop**	3 (14.28)	18 (85.71)	8 (38.09)	13 (61.90)
**Total**	23 (29.87)	54 (70.12)	40 (51.94)	37 (48.05)
***P*-value**	0.06		0.27	

Four patients (7.14%) in straight graft had subclavian double - lumen while only 2 patients (9.5%) in the other group had the same (*P* = 0.9). Seventeen patients in the straight group and 4 patients in the loop group were taking aspirin (*P* = 0.6).

In the straight group, 26 patients (46.42%) experienced thrombosis once versus 8 patients (38.09%) in the loop group (*P* = 0.8), while 25 patients (44.64%) in the straight group suffered from frequent thrombosis versus 8 patients (38.09%) in the loop group. There was no significant statistical difference (*P* = 0.8) in the graft complication rate including steal syndrome, pseudo aneurysm formation, bleeding, venous hypertension, infection, and graft dysfunction rate due to infection. Straight grafts and loop grafts were not significantly different between these two groups (Table 2).

**Table 2:** Compression Between Graft Complication Rate in Straight Grafts and Loop Grafts

Graft Complications	Straight Graft (%)	Loop Graft (%)	*P*-value
**Steal syndrome**	2 (3.57)	2 (9.5)	*P* = 0.341
**Pseudo aneurysm formation**	8 (14.28)	4 (19.04)	*P* = 0.270
**Bleeding**	3 (5.35)	1 (4.67)	*P* = 0.439
**Venous hypertension**	6 (10.71)	2 (9.52)	*P* = 0.892
**Infection**	7 (12.5)	2 (9.52)	*P* = 0.892
**Graft dysfunction due to infection**	4 (7.14)	1 (4.67)	*P* = 0.621

Graft patency was compromised in 31 patients not correlating with infection. Four of them were forced to remove the grafts within 3, 4, 6 months later due to infection. Therefore, infection incidence was 10 (12.98%) among straight grafts versus 9.5% (2 patients) in the other group (*P* = 0.6). Ultimate graft failure was 48.21% (27 patients) and 28.57% (6 patients) in straight grafts and curved ones respectively. Three (5.35%) of them were in the straight graft group but no patients in the loop graft group experienced graft failure in less than a week (*P* = 0.4).

The patency rate in diabetic patients at 1 and 24 months were 91.30% (21 out of 23 patients) and 65.21% (15 patients) respectively; those of the non-diabetic patients were 48 patients (88.88%) and 19 patients (35.18 %) respectively (*P* = 0.4). The patency rate of hypertensive patients at 1 and 24 months were 90% (36 patients out of 40) and 25% (10 patients) respectively, while those of non-hypertensive patients were 94.59% (35 patients out of 37) and 27.02% (10 patients ) respectively (*P* = 0.4).

Venous hypertension occurred in 2 patients out of 6 (33.33%) who received a subclavian double – lumen catheter on the same side whereas it complicated 8.45% of patients without a history of subclavian double – lumen in insertion (*P* = 0.3). Steal syndrome and venous hypertension complicated 6% (3 patients) versus 3.70 % (one patient) and 10% (5 patients) versus 3 patients (11.11%) that used of 6 mm graft and 8 mm one respectively (*P* = 0.2, 0.7 respectively) (Table 3).

**Table 3:** Showing the Primary and Secondary Patency Rate at Different Time Scale a

Graft Complications	Straight Graft (%)	Loop Graft (%)	*P*-value
**Steal syndrome**	2 (3.57)	2 (9.5)	*P* = 0.341
**Pseudo aneurysm formation**	8 (14.28)	4 (19.04)	*P *= 0.270
**Bleeding**	3 (5.35)	1 (4.67)	*P* = 0.439
**Venous hypertension**	6 (10.71)	2 (9.52)	*P* = 0.892
**Infection**	7 (12.5)	2 (9.52)	*P* = 0.892
**Graft dysfunction due to infection**	4 (7.14)	1 (4.67)	*P* = 0.621

^a^Significantly different between two groups

Total primary patency rate were 89.6%, 68.9%, and 56.6% at one, three and six months respectively; those of straight ones were 88.9%, 66.7%, and 51.2 % respectively versus 92.3%, 76.9%, and 75% in loop group which were not significantly different in both (*P* > 0.05). Secondary patency rate in the straight graft group at 12, 18, and 24 months were 57.9%, 52.9%, and 37.9% respectively in contrary to those of the loop ones with 90%, 80%, and 66.7% rate, which were significantly different at 2 years patency rate (*P* = 0.04). Independent association between graft replacement method (straight or loop) and its primary and secondary patency rate was elucidated using logistic regression.

Mean primary patency duration were 11.1 ± 4.1 months and 9.9 ± 6.9 months in straight grafts and loop ones, respectively (*P* = 0.8). Mean Secondary patency duration were 14.5 ± 4.7 months and 13.8 ± 9.5 months in straight grafts and loop ones, respectively with no significant difference (*P* = 0.8). During follow-up visits, 5 patients in the straight groups and two patients in the loop groups were excluded because they died. Mean Duration of Gore-tex replacement to the time of death was 15 months (0.3-36 months), affecting the calculated patency rate. During 2 years serial follow-up, neither vascular access short-time complications nor long-term complications caused mortality.

## 5. Discussion

Long-term patency of arteriovenous fistula in the majority of end stage - renal disease patients has been disappointing. Hence the lack of suitable vascular access causes repeated procedures (7). Ploytetrafluorethylene (PTFE) grafts placement construct a suitable vascular access (10).

In this study, we aim to assess and compare efficacy and complications of straight arteriovenous grafts versus loop ones. There were no significant differences in possible confounding data among two groups. There were several studies noting axillary loop PTFE grafts; all assessed their throacic replacement (11-13). Unfortunately there are few studies against upper arm ones (14). Based on the results presented, we conclude that the short–term patency rate were the same among two groups while the long-term patency rate of loop grafts was higher than that of straight grafts at the 2-year point.

The superiority of loop grafts functionally is definitely independent of possible confounding factors. Mean patency duration were not significantly different in both groups as PTFE loop graft was implanted as an arteriovenous conduit in only one patient during 2004 - 2008 and rest of them were replaced during last 2 years, thereby compromising the long-term follow-up in contrast to the straight ones with 50, 52 and 57 months patency rate follow-up.

The ultimate patency rate of loop grafts, although mostly replaced during last 2 years, was higher than that of straight ones at 2 years. In this study, the primary patency rate of loop grafts was 60% at 1 year, comparable to the 67% of Baron et al. study (14), moreover elucidated the secondary patency rate of 64.6% for upper arm grafts comparable with the results of Mundu *et al*. (15).

The frequency of complications were the same among the two methods of graft replacement and mal incidence of thrombosis, infection, delayed infection, pseudoaneurysm formation and steal syndrome occurrence, ultimate graft failure and venous hypertension were not significantly different. There was no association between co morbid diabetes and hypertension with the short-term and long-term patency rate. Age did not affect the ultimate graft function.

In contrast with the ancient theory of raised steal syndrome occurrence with larger grafts (16), in our study rate of steal syndrome was not associated on the grafts’ size as with Garcia *et al*. study (17). Following placement of a subclavian catheter for even as short a period as 2 weeks, up to 50% of subclavian veins have a significant stenosis that causes either clotting of the PTFE graft or a swollen arm after graft placement (venous hypertension) (16). In the present study, we claimed that patients in both groups had similar rate of venous hypertension or other complications due to ipsilateral subclavian double – lumen catheter.

In this study, two out of six patients with a history of ipsilateral subclavian double – lumen catheter replacement suffered from venous hypertension, and the others faced early graft failure due to near total stenosis of subcalvian vein, clarifying the significant possibility of stenosis (50%) followed by subcalvian double – lumen catheter replacement, which was also indicated in other studies (16)

In a recent study, steal syndrome occurred in a few patients of the loop graft group, possibly boosting the theory of decreased steal syndrome with loop graft replacement or increased length of the graft (14). Ten patients experienced graft infection while it was functional and in two of them it was followed by Gore-tex hematoma. The resulting 40% medical success rate was without graft removal, and in other studies it was reported 25 - 50% (16).

In the present study, in 12.90% of patients who experienced graft-failure due to non-infectious reasons, we removed the graft in 6 months duration since it was complicated later by infection, comparable with the same report in 32 - 40% of immune compromised patients (18, 19). In our study, the loop grafts length were two times higher than straight ones which may have increased the thrombosis formation, but on the other hand, it may explain the superiority of higher secondary patency rate at 2 years in line for restored arterial flow. Furthermore the higher length of loop grafts permit the high afferent-efferent puncture, resulting in significant decrease of recirculation and possibly increasing the dialysis efficacy (14).
